# Carbon-nanotube yarns induce axonal regeneration in peripheral nerve defect

**DOI:** 10.1038/s41598-021-98603-7

**Published:** 2021-10-01

**Authors:** Atsushi Kunisaki, Akira Kodama, Masakazu Ishikawa, Takahiro Ueda, Marcio D. Lima, Takeshi Kondo, Nobuo Adachi

**Affiliations:** 1 Department of Orthopaedic Surgery, Graduate School of Biomedical and Health Sciences, Hiroshima University, Hiroshima, Japan; 2 Nano-Science and Technology Center, LINTEC OF AMERICA, INC., Richardson, USA

**Keywords:** Neuroscience, Neurology, Materials science

## Abstract

Carbon nanotubes (CNTs) are cylindrical nanostructures and have unique properties, including flexibility, electrical conductivity, and biocompatibility. We focused on CNTs fabricated with the carbon nanotube yarns (cYarn) as a possible substrate promoting peripheral nerve regeneration with these properties. We bridged a 15 mm rat sciatic nerve defect with five different densities of cYarn. Eight weeks after the surgery, the regenerated axons crossing the CNTs, electromyographical findings, and muscle weight ratio of the lower leg showed recovery of the nerve function by interfacing with cYarn. Furthermore, the sciatic nerve functional index (SFI) at 16 weeks showed improvement in gait function. A 2% CNT density tended to be the most effective for nerve regeneration as measured by both histological axonal regeneration and motor function. We confirmed that CNT yarn promotes peripheral nerve regeneration by using it as a scaffold for repairing nerve defects. Our results support the future clinical application of CNTs for bridging nerve defects as an off-the-shelf material.

## Introduction

An injured peripheral nerve can regenerate spontaneously or via a microsurgical suture. However, in long-term defects or severe injury, the regeneration capacity of a nerve is limited^1^. Until recently, an interposition autologous nerve graft has been considered the gold standard for nerve regeneration and has produced the best results for peripheral nerve injury with advanced crushing or defects since the first evidence of autologous nerve grafting in animal studies in 1967^2,3^. However, the use of this technique is not without problems, including limited supply, associated donor-site morbidity, and size mismatches with the injured nerve^4^. Several artificial nerve conduits have been tested in clinical applications to overcome these limitations of autologous nerve grafting. However, clinical results are limited, feature low success rates, and are typically only used for less than 3 cm nerve gaps involving small-diameter, non-critical sensory nerves^5,6^.

Therefore, at present, sufficient results cannot be obtained for the treatment of nerve gaps in long or highly functionally critical areas, thus indicating the importance of developing a nerve guide with a stronger therapeutic effect.

This study focused on carbon nanotubes (CNTs) as possible devices to promote peripheral nerve regeneration. In recent years, nanotechnology breakthroughs have advanced the development of new materials, including CNTs. These devices are cylindrical nanostructures, 0.4 to 40 nm in diameter, consisting of interwoven graphene sheets^7^. Due to the unique properties of CNTs, such as strength, flexibility, conductivity, ease of manufacture, and high biocompatibility^8^, this material has been of interest in recent years in biomedical and tissue engineering applications^9^.

In terms of nerve regeneration, several studies have shown that CNTs can support sustainable neuronal survival and promote neuronal outgrowth^10–14^. For hippocampal neurons cultured on a CNT patterned substrate, CNTs were able to direct neurite outgrowth^11^. Aminated CNTs and nerve growth factor (NGF) increase the number of neurons with neurite outgrowth in rat PC12h cell and dorsal root ganglion (DRG) neurons in culture media^15^.

Furthermore, CNTs are ideal for interaction with electrically active tissues, such as neuronal tissues. Neurons that grow on a conductive nanotube meshwork are involved in electrical interactions between CNTs and neurons, which may stimulate neuronal circuits^16^. According to one review article, network hybrids of neurons and nanotubes may predict or manipulate the interactions between nanomaterials and neurons, leading to the design of smart biomaterials for the engineering of electrically propagating tissues^17^.

However, in vivo studies on the impact of CNTs in neuro-regeneration are limited, with only one previous study having been conducted. In this study, nerve conduits filled with fibers incorporating aminated CNTs were chemically tethered onto the surface of phosphate glass microfibers (PGF) and implanted in rat sciatic nerve defects^18^. One reason for the small number of existing in vivo studies is related to the loss of intrinsic properties of individual CNTs that occurs in macroscopic forms of CNTs. Various CNT forms have been demonstrated to overcome this matter, including fibers^19,20^ yarns^21,22^. Therefore, we aimed to evaluate the efficacy of CNTs in peripheral nerve regeneration in vivo using the processed CNT yarns as the intraluminal fibers of artificial nerve conduits.

We investigated whether regeneration of peripheral nerves was promoted by transplanting CNT yarn into the nerve defects as a scaffold to explore the possibility of using CNT yarn as a novel artificial nerve material.

## Methods

Methods of this study were reviewed and approved by the ethical committee of Hiroshima University. And all experiments were performed in accordance with relevant guidelines and regulations. This study was also carried out in compliance with the ARRIVE guidelines.

### Production of the implant

**Figure 1:**
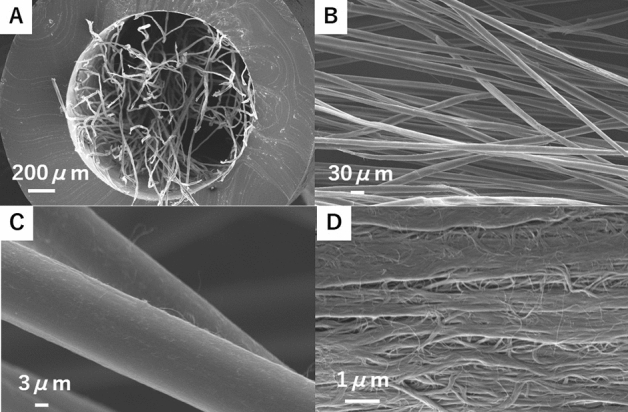
Morphology analysis using scanning electron microscopy of carbon nanotube fibers embedded in a silicon tube. (**A**) The whole cross-sectional structure; with cYarn fibers inside the silicon tube at a 2% density. (**B**) Magnified surface of aligned fibers structures inside the silicon tube. (**C**) External surface of cYarn fibers. (**D**) Magnified external surface of cYarn fibers.

The CNT yarn used in this study was cYarn (LINTEC of AMERICA INC., Nano-Science & Technology Center, Richardson, TX, USA) manufactured through dry drawing and spinning^23–26^. As the CNT source, vertically aligned multiwall CNT (MWCNT) forests produced by chemical vapor deposition using acetylene as carbon source and iron as catalyst were used. The typical diameter and length of MWCNT were approximately 10 nm and 300 μm, respectively. Yarns were approximately 15 μm in diameter. For in-vivo tests, CNT yarns were inserted in silicon tubes with a lumen of 15 mm (Fig. 1).

### Experimental animals and surgical procedures

Thirty-five female Sprague Dawley rats (8 weeks old and weight: 200–220 g) were used in this investigation. Rats were housed in groups of three animals under standard conditions. For anesthesia, Rompun (20 mg/ml, BAYER HEALTH CARE, Leverkusen, Germany) and Ketalar (50 mg/ml, DAIICHI-SANKYO, Tokyo, Japan) were injected intraperitoneally for each animal. The rat sciatic nerve defect model was induced as previously described^27,28^. A skin incision was made along the lateral femur, expose the sciatic nerve by dissection of the gluteus and biceps femoris muscles. The sciatic nerve was transected at the middle of the thigh with microscissors. For CNTs transplantation, 10 mm of the sciatic nerve was removed. Just after the nerve injury, 1 mm of both ends of the transected sciatic nerve were inserted into 17 mm silicon tubes filled with 15 mm CNT yarn fibers to bridge the nerve defect. In other words, a 15 mm nerve defect was made. Previous studies have demonstrated that the rat 15 mm sciatic nerve defect model does not spontaneously regenerate nerves^27,28^. Therefore, in this study, we used a 15 mm sciatic nerve defect model as a negative control, in which a silicon hollow tube was transplanted into the defect. Before implantation, silicon tubes and CNT yarns were washed in NaCl after autoclave sterilization. The proximal and distal nerve stump and tubes were connected by three sutures using 8–0 monofilament nylon thread.

### Experimental design and groups

In order to analyze the performance of different densities of CNT fibers in vivo, we created five experimental groups (C0: control group, silicon hollow tube; CN2: CNT yarn filling at a low density of 2% [200 bundles] in the silicon tube; CN5: moderate density of 5% [500 bundles]; CN10: high density of 10% [1000 bundles]; CN35: high density of 35% [3500 bundles]).

Two different observation periods were chosen after which the animals were finally examined and sacrificed prior to the explanation of the implants and regenerated tissue: as short term periods eight weeks (C0, CN2,CN5,CN10,CN35, n = 7 animals in each group), as long term period 16 weeks (C0 n = 6, CN2 n = 7, respectively). The motor recovery was evaluated by calculation of sciatic nerve functional index (SFI) every four weeks and electromyography before sacrifice animals. The examiner was blinded to the implants for sciatic nerve reconstruction applied to each animal. All rats were euthanized with 100% carbon dioxide inhalation after an overdose of Rompun and Ketalar by intraperitoneal injection at the conclusion of the electrophysiological studies.

The sciatic nerves along with the silicon tubes and CNT fibers were harvested 8 weeks and 16 weeks after the surgery. The silicon tubes were removed, and immediately afterward the contents inside the silicon tube with proximal and distal nerve segments were embedded in OCT Compound (TISSUE-TEK, SAKURA FINETEK, Tokyo, Japan). In the 8-week evaluation, the nerve was sectioned longitudinally at 8 µm thickness and in the 16-week evaluation, proximal and distal nerve segments were sectioned transversely at 8 µm thickness. Sectioning was performed in accordance with a method described previously^29^. The sectioning surface was covered with an adhesive film (CRYOFILM TYPE IIC9, SECTION-LAB, Hiroshima, Japan) and frozen sections were made with a microtome (CRYOSTAT HM520, THERMO FISHER SCIENTIFI K.K., Tokyo, Japan). The resulting sections were stained with hematoxylin and eosin (H&E) and immunohistochemistry analysis using a BZ-9000 microscope (KEYENCE CORP., Osaka, Japan).

### Electrophysiological study

Electrophysiological examination was performed according to the method of our facility previously described^28^. Briefly, the sciatic nerves proximal to the silicone tubes were exposed, and needle electrodes were placed in the gastrocnemius muscle. The nerves were stimulated with a constant current of 2.0 mA (0.2 ms square-wave pulses) using bipolar electrodes. The stimuli were applied to the sciatic nerve proximal to the silicone tube at the experimental side and to the sciatic nerve at the same level as the contralateral side. The compound muscle action potentials (CMAPs) were recorded after stimulation using the Viking Quest system (NICOLET BIOMEDICAL, Madison, WI, USA). The onset latency and peak to peak amplitude of the CMAPs from the experimental side were compared with those recorded from the contralateral side.

### Muscle weight ratio

After the animals were sacrificed, the gastrocnemius muscle and tibialis anterior muscle were excised from bilateral hindlimbs and weighed to calculate the ratio of the experimental side compared to the contralateral side.

### Immunohistochemistry

Immunohistochemistry was performed, with neurofilament (NF) antibodies marking axons and S100 antibodies identifying Schwann cells. Briefly, the section was fixed in a 4% paraformaldehyde and methanol mixture for 30 s and washed with cold PBS three times for 3 min each time, and then blocked with 10% normal goat serum (LIFE TECHNOLOGY CORP., Carlsbad, CA, USA) at 4 °C for 1 h. Following this, immunostaining was performed with anti-chicken NF protein (ABCAM, Cambridge, UK), diluted 1:200 in PBS and anti-rabbit S100 protein (ABCAM) diluted 1:200 in PBS.

The second day, the sections were washed three times with PBS, then incubated with the secondary antibody Alexa Fluor 488 goat anti-Rb IgG and Alexa Fluor 568 anti-chicken IgG (THERMO FISHER SCIENTIFIC K.K.), diluted in 1:200 in PBS, and coverslipped with DAPI counterstain for 1 h at room temperature. In addition, another immunohistochemistry was performed with CD68 antibodies marking pan-macrophage. Briefly, immunostaining was performed with anti-mouse CD68 protein (ABCAM), diluted 1:100 in PBS. After incubated overnight at 4 °C, then incubated with the secondary antibody Alexa Fluor 350 anti-mouse IgG (THERMO FISHER SCIENTIFIC K.K.), diluted in 1:200 in PBS for 1 h, and cover slipped with DRAQ5 (ABCAM) counterstain for 5 min at room temperature.

The photomicrographs of these sections were taken using a fluorescence microscope (KEYENCE, Osaka, Japan) which connect to a digital camera and computer.

### Image analysis

In the longitudinal sections of the 8-week evaluation, the immunoreactivity (IR) of neurofilament, number of S100 positive cells was measured at four areas (P, proximal nerve end; P5, 5 mm from the proximal stump; D5, 5 mm from the distal stump; and D, distal nerve end) in three randomly selected sections. In the cross-sections of the 16-week evaluation, the numbers of neurofilament, the numbers of S100 positive cells and the IR of S100 was measured at the proximal and distal nerve end. The immunoreactivity was calculated using the ratio of the immunoreactivity area at the P5, D5, and D nerve sites to the P site, referring to the previously described methods^30,31^. For cell count, the numbers of S100 stainable structures with DAPI stained nuclei were counted respectively by manual cell counting. The numbers of CD68 stained multinucleated cells showing activated macrophages were counted at the area of P5 in the longitudinal section to evaluate foreign body reaction to CNT. The analyzing area of each section was 800 × 600 μm. And the magnification of the image was 400x. The ImageJ software (NATIONAL INSTITUTE OF HEALTH [NIH], Bethesda, MD, USA) were used for the analysis.

### Statistical analysis

Data are presented as the average ± standard error (SE). One-way ANOVA with post-hoc Tukey–Kramer test was used to determine the differences between groups for CMAPs, muscle weight ratio, and immunohistochemical evaluation in 8 weeks evaluation. Student t-test was used to compare the two groups (C0, CN2) in the 16 weeks evaluation. Statistical significance was established at p < 0.05.

## Results

### Macroscopic findings

**Figure 2:**
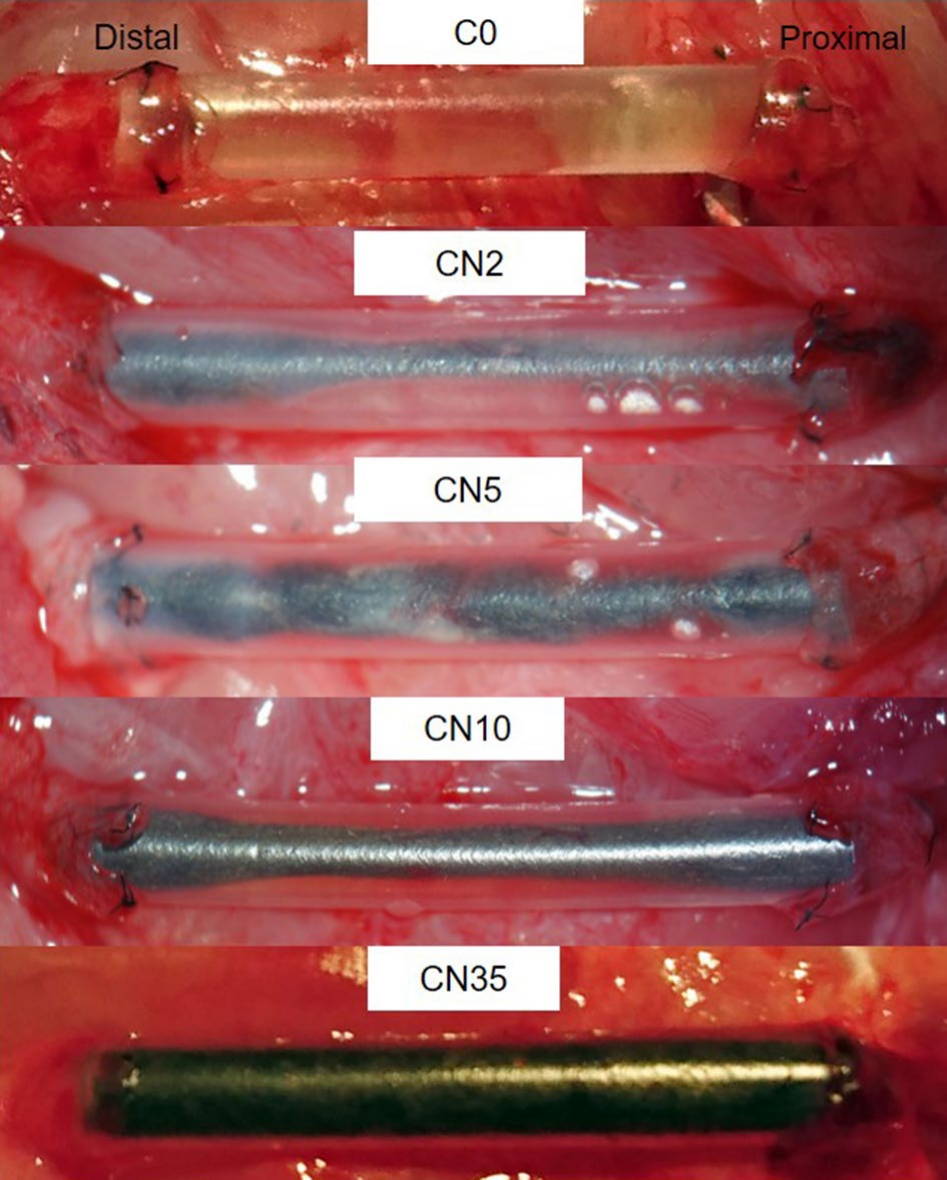
Intraoperative photographs showing the macroscopic appearance of the regenerated tissue along with the carbon nanotube yarns eight weeks after transplantation.

8 weeks after the surgery, five rats did not exhibit any continuity in defect bridging, and two rats exhibited extremely poor continuity with scar-like tissue in the control group. In three of the four groups, those filled with a moderate density of CNT yarn (CN2, CN5, and CN10), the CNT yarn gathered to form one cord. On the surface, white tissue and newly formed capillary vessels were observed. However, tissue formation was not observed around the CNTs macroscopically in the CN35 group (Fig. 2).

### Electrophysiological evaluation

In 8 weeks, CMAPs were detected in six of seven rats in each of the CN2 and CN5 groups, and three of seven rats in the CN10 group. The mean latency and the mean amplitude were 4.96 ± 0.63 ms and 4.75 ± 1.05 mV in CN2; 4.95 ± 0.75 ms and 3.08 ± 0.27 mV in CN5; and 5.38 ± 1.24 ms and 4.44 ± 1.18 mV in CN10 respectively. However, no CMAPs were obtained in the C0 or CN35 groups. No significant difference in the latencies and amplitudes between CN2, CN5, and CN10 groups were observed. In 16 weeks, CMAPs were detected six of seven rats in the CN2 groups. However, no CMAPs were obtained in the C0 group (Table 1).

**Table 1:** The compound muscle action potentials of the gastrocnemius for the five test groups at 8 and 16 weeks after transplantation.

Group (number)		C0	CN2	CN5	CN10	CN35
Number (8 Wks/16 Wks)		(7/6)	(7/7)	(7/–)	(7/–)	(7/–)
Recovery rate (%)	8 Wks	0 (0/7)	85.7 (6/7)	85.7 (6/7)	42.9 (3/7)	0 (0/7)
	16 Wks	0 (0/6)	85.7 (6/7)			
NCV (ms ± SE)	8 Wks	–	4.96 ± 0.63	4.95 ± 0.75	5.38 ± 1.24	–
	16 Wks	–	3.87 ± 0.6			
NCV ratio (contralateral/ipsilateral)	8 Wks	–	0.62 ± 0.1	0.57 ± 0.10	0.46 ± 0.14	–
	16 Wks	–	0.52 ± 0.1			
Peak amplitude (mV ± SE)	8 Wks	–	4.75 ± 1.05	3.08 ± 0.27	4.44 ± 1.18	–
	16 Wks	–	6.02 ± 2.6			
Peak amplitude ratio (ipsilateral/contralateral)	8 Wks	–	0.52 ± 0.1	0.43 ± 0.07	0.52 ± 0.1	–
	16 Wks	–	0.43 ± 0.2			

*NCV* nerve conduction velocity, *SE* standard error.

### Muscle weight ratio

Muscle weight ratio was expressed as % of the contralateral side. The tibialis anterior muscle weight ratio was significantly greater in the CN2 and CN5 groups compared to the C0 group. The ratios of the gastrocnemius in the CN2, CN5, and CN10 groups were also greater than that of the C0 group (Table 2).

**Table 2:** Lower limb muscle weight ratio for the five test groups at 8 and 16 weeks after transplantation.

Muscle weight ratio (% ± SE)		C0	CN2	CN5	CN10	CN35
Tibialis anterior	8 Wks	23 ± 1.24	32.6 ± 3.5*	31 ± 2.4 *	28 ± 3.0	23.8 ± 1.57
	16 Wks	19.5 ± 1.36	39 ± 3.9**			
Gastrocunemius	8 Wks	20.1 ± 1.18	33 ± 4.4**	29.6 ± 2.6**	28.6 ± 1.9*	21.1 ± 1.2
	16 Wks	17.8 ± 1.37	39.8 ± 4.7**			

*P < 0.05; **P < 0.01 (One-tailed ANOVA with Tukey–Kramer test (8 weeks) and Student t-test (16 weeks) compared with C0).

### Sciatic nerve functional index (SFI)

**Figure 3:**
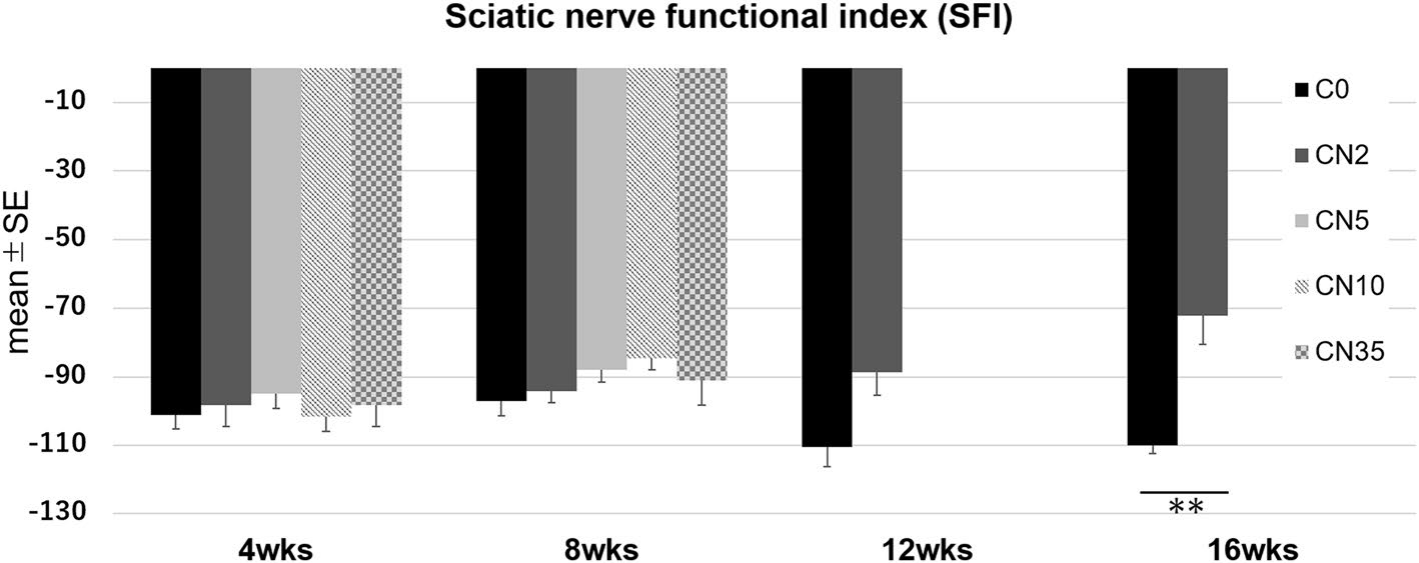
Results of the Sciatic nerve functional index (SFI). *** P < 0.001 (One-tailed ANOVA with Student t-test compared with C0).

There was no significant difference in the sciatic nerve functional index (SFI) between any of the groups at 4 or 8 weeks after surgery. However, at 16 weeks, SFI increased significantly in the CN2 group (− 72 ± 8.5) compared to the C0 group (− 110 ± 2.2) (Fig. 3).

### Histological and immunohistochemical evaluation for axonal regeneration of excised sciatic nerve

**Figure 4:**
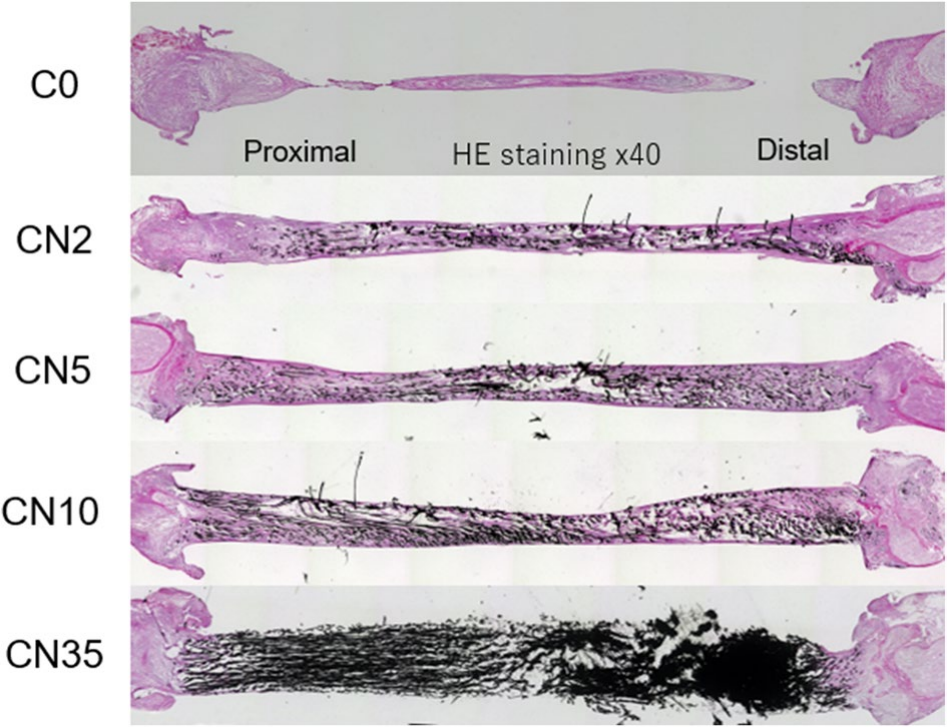
Representative light micrograph of longitudinal section of the carbon nanotube nerve guide eight weeks after sciatic injury and repair with hematoxylin and eosin staining.

In the 8-week evaluation, the H&E staining showed that the nerve defect was crosslinked with the regenerated tissue in all cases found in the CN2, CN5, and CN10 groups. However, no meaningful tissue regeneration occurred in the C0 or CN35 groups (Fig. 4).

**Figure 5:**
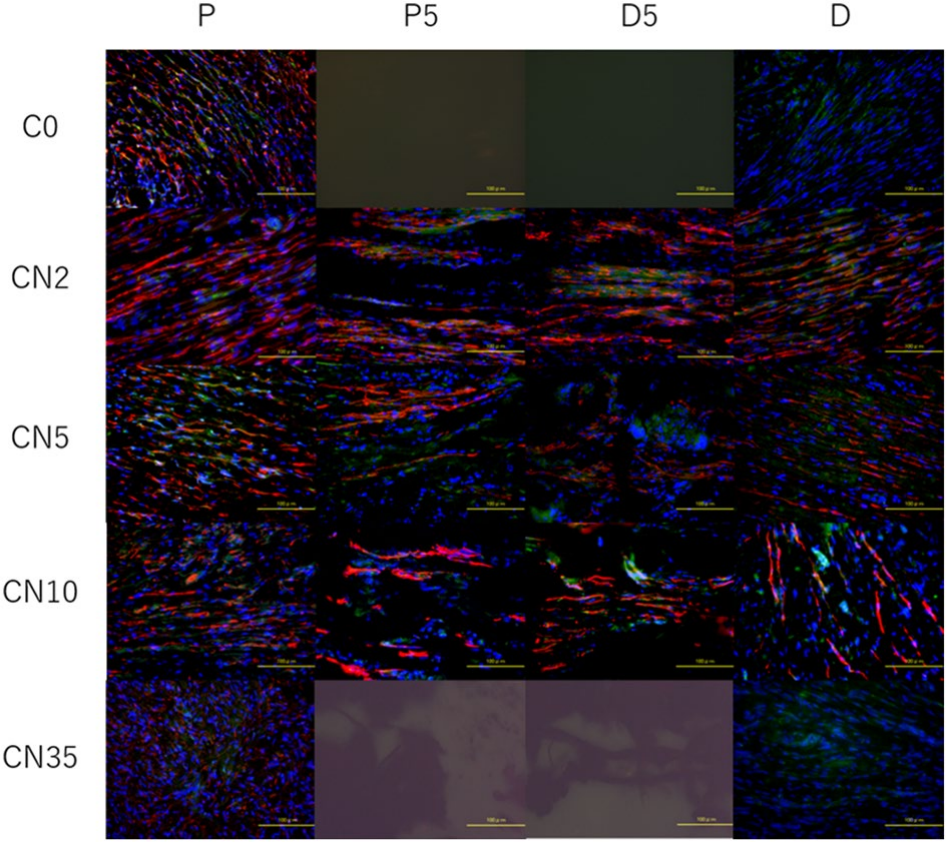
Longitudinal sections of the carbon nanotube nerve guide eight weeks after implantation with immunohistochemical staining. Sections were stained with neurofilament (axons, red), S100 (Schwann cells, green) and DAPI (cell nuclei, blue). Four areas (P: proximal nerve end; P5: 5 mm from the proximal stump; D5: 5 mm from the distal stump; D: distal nerve end) of each section were observed. Scale bar indicates 100 µm.

Triple immune fluorescence provided a visualization of nerve regeneration, in which longitudinal sections of the regenerated tissue were stained using S100 and NF. The ratio of NF immunoreactivity at the distal end was higher in the CN2 and CN5 groups than in the C0 group (CN2: 62.61 ± 5.37%; CN5: 67.65 ± 9.63%). However, there was no significant difference between the CN2 and CN5 groups. In the CN10 group, neural tissue was observed along cYarn fibers; however, the ratio of NF immunoreactivity at the distal end was lower than in the CN2 or CN5 groups (CN10: 14.85 ± 7.48%). No neurofilament was observed at the distal nerve end in the C0 group, and no regeneration of nerve defect tissue was seen in the CN35 group. There was no statistically significant difference in the number of S100 positive cells at the proximal and distal nerve ends between the groups. The numbers of cells were significantly increased in areas P5 and D5 (within the nerve defect) in the CN2 and CN5 groups compared to the CN10 group. In the C0 group, only a few cells were observed in two of seven rats, and in the CN35 group, no tissue regeneration was observed (Fig. 5, Table 3).

**Table 3:** Immunohistochemical evaluation of axonal outgrowth and number of S100 positive cells in the matrix and in the distal nerve segment of rat sciatic nerve defects, bridged by different densities of carbon nanotube yarn in 8 weeks after the transplantation.

	C0 (n = 7)	CN2 (n = 7)	CN5 (n = 7)	CN10 (n = 7)	CN35 (n = 7)
**IR of Neurofilament (% of proximal portion)**					
P5/P	8.9 ± 6.9	95.7 ± 12.2***	87.6 ± 11.7***	56.6 ± 12.0**	0 ± 0
D5/P	0.2 ± 0.2	72.9 ± 15.3***	84.9 ± 10.8***	19.4 ± 7.9*	0 ± 0
D/P	0 ± 0	62.6 ± 5.4***	67.7 ± 9.6***	14.9 ± 7.5	0 ± 0
**Number of S100 + cells (% of proximal portion)**					
P5/P	19.7 ± 12.6	77.9 ± 7.3**	63.5 ± 9.8*	55.3 ± 8.9	0 ± 0
D5/P	0 ± 0	69.0 ± 17.6**	57.1 ± 4.3*	31.7 ± 7.9	0 ± 0
D/P	77.3 ± 11.6	95.6 ± 20.1	73.7 ± 8.0	82.3 ± 6.7	57.4 ± 10.8

*IR* immunoreactivity, *P* proximal nerve end, *P5* 5 mm from the proximal stump, *D5* 5 mm from the distal stump, *D* distal nerve end.

*P < 0.05, ** P < 0.01, *** P < 0.001 (One-tailed ANOVA with Tukey–Kramer test compared with C0).

**Figure 6:**
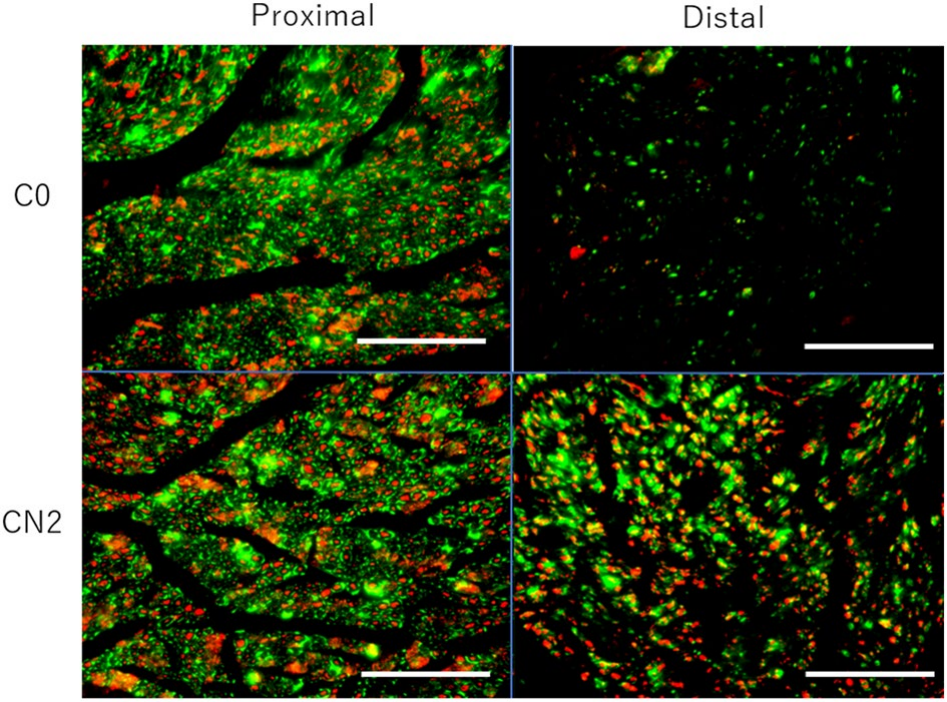
Transvers sections of the proximal and distal nerve segments of the C0 and CN2 groups 16 weeks after implantation. Sections were stained with neurofilament (axons, red), S100 (Schwann cells, green). Scale bar indicates 100 µm.

In the 16-week evaluation, the number of S100 positive cells and neurofilaments at the distal end were significantly higher in the CN2 group than in the C0 group. (Fig. 6, Table 4).

**Table 4:** Immunohistochemical evaluation of axonal outgrowth, number of S100 positive cells and immunoreactivity of S100 in the proximal and the distal nerve segment in 16 weeks after the implantation.

		C0 (N = 6)	CN2 (N = 7)
Number of NF	Proximal (no/mm^2^)	9081 ± 815	8600 ± 594
	Distal (no/mm^2^)	756 ± 114	8242 ± 619***
	Distal/Proximal (%)	8.5 ± 1.1	96.0 ± 3.0***
Number of S100 + cells	Proximal (no/mm^2^)	9475 ± 897	8821 ± 596
	Distal (no/mm^2^)	2556 ± 566	8425 ± 633***
	Distal/proximal (%)	28.7 ± 7.4	95.5 ± 3.0***
IR of S100	Proximal (%)	21.4 ± 2.1	25.0 ± 1.4
	Distal (%)	5.1 ± 1.2	20.1 ± 1.3***
	Distal/proximal (%)	26.4 ± 7.6	81.3 ± 3.98***

*NF* neurofilament, *D/P* Distal/Proximal, *IR* immunoreactivity.

*** P < 0.001 (Student t-test compared with C0).

### Immunohistochemical evaluation for foreign body reaction

**Figure 7:**
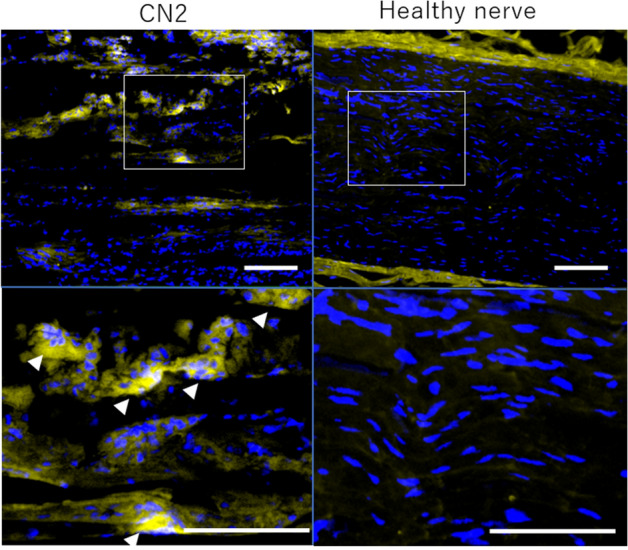
Longitudinal sections of the regenerated tissue 5 mm from the proximal stumps of the C0 and CN2 groups 16 weeks after implantation. Sections were stained with CD68 (Macrophages), DRAQ5 (cell nuclei, blue). Scale bar indicates 100 µm. White arrows indicate CD68 positive multinuclear giant cell.

CD68-positive cells in the regenerated tissue 5 mm from the proximal stump were measured in the longitudinal section. CD68-positive cells were found in the CN2 group at 156.9 (SE22.5)/mm^2^. On the other hand, these cells were hardly found in healthy nerves (n = 4). (Fig. 7).

## Discussion

This study demonstrated successful regeneration of peripheral nerves using CNT yarn as a nerve scaffold in vivo by anatomical as well as functional measures for the first time. In contrast, the 15 mm nerve defect did not show spontaneous reconstruction within a hollow silicone tube as described in the previous studies^27,28^. These results indicate that the nano-scale topographical scaffold alone enabled significant regeneration, with no exogenous neurotrophic proteins nor cell transplantation. The sagittal section of the immunohistochemical analysis indicated that, across the distance of the transected sciatic nerve, axons extended along with the aligned CNT yarns with migrated Schwann cells and reached the distal stump. Electrophysiological and muscle weight analysis also demonstrated that the CNT constructs facilitated motor nerve regeneration and significantly improved the functional deficit after the peripheral nerve injury. Our results indicated that a 2% CNT density tended to be most effective for nerve regeneration as measured by both histological axonal regeneration and motor function. We consider that tissue regrowth did not occur at the 35% CNT density due to the high occupancy of CNT yarns in the silicon tube, which may have impeded the perfusion of tissue fluid. In addition, 16 weeks long term evaluation was performed in the 2% CNT group which was the optimal outcome in the eight weeks evaluation, and an evident functional recovery including SFI was observed as compared with the hollow silicon tube group.

This study confirmed the effect of CNTs on peripheral nerve regeneration in vivo and clarified the optimum density of CNT fiber scaffold.

However, other biological impacts of CNT yarns, including effects on cell migration ability and cell adhesion, were not elucidated. Further investigations of peripheral nerve regeneration mechanisms alongside this material are needed before more efficient regeneration protocols can be achieved.

In nerve conduits of various materials, it is important that a fibrin matrix forms to bridge the proximal and distal nerve stumps. The fibrin matrix contains inflammatory cells and vascular endothelial cells, and its formation is crucial for the migration and proliferation of Schwann cells and axonal growth. The formation of the fibrin matrix is facilitated by the tube structure to a certain extent, as it connects the proximal and distal nerve ends^32,33^. However, If the distance between the nerve ends is too long, the matrix is not formed and the regeneration process across the nerve defect is interfered^34,35^.

Recent studies have revealed the mechanism by which two nerve stumps reconnect during nerve repair. First, macrophages secrete VEGF-A, which stimulates the formation of blood vessels oriented in the direction of nerve regeneration. The Schwann cell cord (Büngner band) is then formed using the polarized blood vessels as a migratory scaffold. Finally, axons extend from the proximal stump to the distal stump, guided by the Schwann cell cord^36^. In support of these known mechanisms, the intraluminal fibers may induce fibrin cable polarized blood vessels and Schwann cell cords and thus help to bridge the proximal and distal nerve stump over long nerve gaps.

A study culturing rat hippocampal neurons on two patterns of CNT yarns (parallel aligned and cross linked) demonstrated that almost all neurites grow along the CNT yarns in the growth direction of the neurites. Even on the cross-linked CNT yarn patterned substrate, a neurite could grow along one CNT yarn and then turn towards another cross-linked yarn. This indicates that CNT yarns possess the main characteristics of a guidance scaffold for neurite outgrowth^37^.

Another possible reason why nerve tissue was regenerated along the cYarn fiber is thought to be the CNT character itself and the structural features of our nerve conduit model composed of thin and aligned fibers. A previous study cultured DRG on different diameters of fiber scaffolds and found that the direction and extent of neurite extension and Schwann cell migration from DRG explants was influenced significantly by fiber diameter^38^. Another study indicated that fibers with smaller diameter have better cell adhesion effects^39^. In addition, Kim et al. demonstrated that aligned, oriented fibers accelerated DRG outgrowth in vitro and nerve regeneration in vivo compared with randomly oriented fibers^40^. cYarn fiber is a fiber of 15 μm in diameter, composed of 10 nm fibers. As shown in Fig. 1, the cYarn surface exhibits irregularities due to these 10 nm fibers, therefore increased surface fiber area. Scaffolds with a high surface area are advantageous for cell adhesion and proliferation^41^.

Although we were able to demonstrate the effectiveness of CNT yarns in peripheral nerve regeneration, this study has several limitations. Firstly, we used an artificial nerve model that combines a silicon tube and CNT yarn, which is far from clinical use. In clinical applications, conduits should be made from biodegradable material. However, in previous in vivo studies examining the effects of nerve guides or cell therapy on nerve regeneration, hollow silicone tubes were inserted as nerve guide in a standard model^42,43^, in order to obtain external stability to provide space for nerve regeneration to occur.

CD68 positive cells that are recognized as macrophages were observed in the regenerated tissue 16 weeks after the CNT yarn implantation. An undeniable foreign body reaction occurred by transplanting the CNT yarn into living animals. The biocompatibility of CNT yarn is desired to be improved by chemical modification or protein bonding in future study. In this study, we demonstrated only the superiority of CNT yarn over the negative control using the silicone hollow tube. The functional results of muscle weight and SFI are inferior to those of healthy nerves even when CNT yarn is scaffolded. For comparison with autografts and decellularized nerve allografts for clinical use, significant superiority would have to be demonstrated. Finally, we succeeded nerve regeneration in a rat 15 mm sciatic nerve defect. However, to prove clinical efficacy for 30 mm or more defects, we need further studies with a long gap in larger animals.

Currently, animal-related research to promote nerve regeneration by stem cell transplantation is underway. However, stem cell transplantation requires cell harvesting and culturing, causing the transplant surgery to be long in duration, with an associated high cost. On the other hand, CNTs have sterilizable and stable material properties, are mass-producible, and can be provided at a low cost. Therefore, CNT artificial nerves may one day be an off-the-shelf product that is unlimited in supply.

Another biological benefit of CNTs is the ease with which a broad range of molecules can be bound to the yarn, due to the extremely high reactivity of the CNT surface^44^. Chemical modifications can be made to increase the probability of nerve regeneration, including modification of electrical charge^11^ and binding of growth factors^45^.

## Conclusions

We confirmed that CNT yarn, used as a scaffold for repairing 15 mm nerve defect in rat sciatic nerve, promotes peripheral nerve regeneration in motor function and histology. Our findings point to 2% CNT yarns as the optimal density for peripheral nerve regeneration. Although CNT yarns require further development in terms of therapeutic effect and biocompatibility, the results of this study support the future clinical application of CNTs as an off-the-shelf material for artificial nerve conduits.
